# Persistence of SARS-CoV-2 total immunoglobulins in a series of convalescent plasma and blood donors

**DOI:** 10.1371/journal.pone.0264124

**Published:** 2022-02-24

**Authors:** M. Carmen Martin, Ana Jimenez, Nuria Ortega, Alba Parrado, Isabel Page, M. Isabel Gonzalez, Lydia Blanco-Peris

**Affiliations:** Centro de Hemoterapia y Hemodonacion de Castilla y Leon, Valladolid, Castilla y León, Spain; University of Pittsburgh, UNITED STATES

## Abstract

**Background:**

The vast majority of COVID-19 cases both symptomatic and asymptomatic develop immunity after COVID-19 contagion. Whether lasting differences exist between infection and vaccination boosted immunity is yet to be known. The aim of this study was to determine how long total anti-SARS-CoV2 antibodies due to past infection persist in peripheral blood and whether sex, age or haematological features can influence their lasting.

**Material and methods:**

A series of 2421 donations either of SARS-CoV-2 convalescent plasma or whole blood from 1107 repeat donors from January 2020 to March 2021 was analysed. An automated chemiluminescence immunoassay for total antibodies recognizing the nucleocapsid protein of SARS-CoV-2 in human serum and plasma was performed. Sex, age, blood group, blood cell counts and percentages and immunoglobulin concentrations were extracted from electronic recordings. Blood donation is allowed after a minimum of one-month post symptom’s relapse. Donors were 69.7% males and their average age was 46. The 250 donors who had later donations after a positive one underwent further analysis. Both qualitative (positivity) and quantitative (rise or decline of optical density regarding consecutive donations) outcomes were evaluated.

**Results and discussion:**

In 97.6% of donors with follow-up, anti-SARS-CoV-2 protein N total antibodies remained positive at the end of a follow-up period of 12.4 weeks median time (1–46, SD = 9.65) after the first positive determination. The blood group was not related to antibody waning. Lower lymphocyte counts and higher neutrophils would help predict future waning or decay of antibodies. Most recovered donors maintain their total anti-SARS-CoV-2 N protein antibodies for at least 16 weeks (at least one month must have been awaited from infection resolution to blood donation). The 10 individuals that could be followed up longer than 40 weeks (approximately 44 weeks after symptom’s relapse) were all still positive.

## Introduction

After one year of the COVID-19 pandemic, one of the most addressed issues about SARS-CoV2 immunity is how long it will last. The vast majority of COVID-19 cases both symptomatic and asymptomatic will develop either antibodies, cell immunity or both after contagion [1,2]. Although evidence exists of SARS-COV-2 infection inducing long-lived bone marrow specific plasma cells even in mild cases [3], it’s not yet established whether infection or vaccination boosted immunity will last as long. The answers to this question would determine key issues such as the reliability of individual and herd immunity or the need of sanitary restrictions or periodical revaccination.

Antibody assays are not equivalent: they either detect antibodies against different viral proteins (S1, S1/S2, RBD or NC) or different immunoglobulin classes: IgG, IgM, IgA or their combinations. Many factors can influence test performance, including cross-reactivity with other coronaviruses that can occur in up to 28% of individuals [1] or platform (laboratory-based vs point-of-care, lateral flow). Chemiluminescence assays have suitable performances regarding both specificity and sensitivity and correlate with titres of neutralizing antibodies [4].

Antibody responses against viral S and N proteins are equally sensitive in the acute phase of infection, but antibody responses against N seem to wane in the post-infection phase whereas those against the S protein would persist over time with a slow decay [3,5]. Both anti N and anti-S antibodies correlate neutralizing antibodies [6]

A typical humoral response with the early expansion of antibodies along the first-month post symptoms’ onset, a contraction and a memory phase following natural SARS-CoV-2 infection would be expected [7]. Even if antibody levels waned, long-lived mononuclear blood cells (MBCs) and long-lived bone marrow plasma cells would remain to mediate rapid antibody production triggered by a new contact with the virus. Some studies suggest that SARS-CoV-2 infection strengthens pre-existing broad coronavirus protection through S2-reactive antibodies and MBCs formation [8]

Antibody evolution surveys are useful to monitor the pandemic, both before and after vaccination strategies [9]. There are several features that should be taken into account, including time-lapse between infection and antibody development or antibody waning. Blood donors constitute a representative subset of the population aged 18–65 and are reasonably free of biases that could over-represent symptomatic or exposed individuals.

Heterogeneity of susceptibility and transmission is hard to evaluate [10]. A portion of the population may have pre-existing immunity via cross-reactivity or particular host factors such as mucosal immunity or trained innate immunity protection (as it has been reported to be conferred by Diphtheria-pertussis-tetanus (DPT) or Bacillus Calmette–Guérin (BCG) vaccination [11]. There is as well a proportion of post-infection seronegative individuals that develop immunity by T cell-mediated responses but without exhibiting an antibody response [12].

To date, it is not possible to ascertain whether antibodies can last longer than the time COVID-19 has been among us. There are recently described early cases in France [12]. The recording of the first cases in Spain began in January 2020. Our first positive donations are from 2019, possibly due to cross-reaction to previous coronaviruses [13] but we decided to start this series in January 2020, after WHO issued a comprehensive package of technical guidance online with advice to all countries on how to detect, test and manage potential cases.

Our starting hypothesis was that both recovered and asymptomatic COVID-19 cases would develop and keep antibodies against the novel coronavirus [1,2,11]. This would have two main implications: (a) both of them will subsequently contribute to herd immunity and antibodies due to prior infection, should be considered regarding vaccination, so far cellular responses of previously infected individuals could even result impaired [14,15].

The main aim of this study was to determine which percentage of the seropositive population due to natural infection keeps total antibodies recognizing SARS-CoV2 and for how long. A secondary goal was to establish whether sex, age, blood group or haematological features might influence seropositivity or how long it will last. This knowledge is relevant to optimize immunization strategies, and to make decisions about the need for periodical revaccination either overall or for determined population groups.

## Materials and methods

The 120,082 donations from 77,259 donors collected from 13/01/2020 to 03/03/2021 were systematically randomized to select a minimum of 127 donations per week, calculated on the basis of the total number of donations and the length of the analysis period. 13,795 samples from 12,741 donors were analysed for a seroprevalence study [13]. 2,432 donation samples came from 1107 repeat donors. The 250 donors with at least two analysed donations, the first one positive, and their 588 donations underwent further analysis. Plasma-EDTA and serum frozen samples from blood, plasma (normal and convalescent) and platelet donations were tested. The infectious status was only known for convalescent plasma donors, but it is difficult to ascertain in any other donors so far asymptomatic, not traced cases or early cases without PCR confirmation test are frequent. Total immunoglobulins anti-SARS-CoV-2 and haematological features were studied in donations including convalescent plasma from repeat donors from January 2020 to March 2021. Given the dates when samples were collected, none of the individuals was vaccinated.

All haematological and demographic data were extracted from our electronic database eDelphyn (Hemasoft). Variables analysed included age, sex, blood group, and laboratory data: leukocyte (WBC), neutrophil, lymphocyte, platelet, monocyte, eosinophil and basophil counts (cells*103/μL) and their percentages, serum immunoglobulins IgG, IgA and IgM (mg/dL), haemoglobin (Hb), and haematocrit (HCT) were analysed as well. This dataset is available as S1 Table in S1 File.

An automated chemiluminescence double-antigen sandwich immunoassay for the in vitro semi-quantitative detection of total antibodies to SARS-CoV-2 in human plasma and serum frozen samples. The target antigen of this immunoassay is a recombinant nucleocapsid (N) protein. Elecsys® Anti-SARS-CoV-2 (Roche, Basel, Switzerland‎) detects antibodies correlating with virus-neutralizing ones and is therefore useful to help characterize the immune reaction to SARS-CoV-2 [6,11,12]. Immunoassay was validated as described in [13]. The cut-off was that recommended by the manufacturer (OD>1 to report reactivity). Sensitivity after 14 days of infection is 100% (88.1–100) and overall specificity 99.81% (99.65–99.91%). Researchers performing anti-SARS-CoV-2 analyses were blind to the condition of COVID-19 convalescence and to any other characteristic of the donors or to the donation dates.

Both quantitative and qualitative changes in antibodies were assessed. We used the word ‘wane’ to designate these cases who are first positive and afterwards negative and ‘keep’ for the ones with only positive results. A total of 340 pairs of consecutive donations were evaluated. The term ‘stay’ in this paper is used for consecutive donation pairs where the second donation yields an optical density (OD) within 90%-110% of the first one; ‘rise’ for those over 110% OD, and ‘decay’ or ‘decline’ for the ones with a second OD under 90% of that of the first donation.

Demographic and clinical characteristics of patients are expressed as their mean, median, standard deviation (SD) and interquartile range (IQR) for continuous variables and frequency distributions are reported for categorical variables. Combined scatter/ box plots (Figs 2 and 5) represent both individual cases and their percentiles 10, 25, 50, 75 and 90 (low whisker, low box side, centre, upper box side, upper whisker). Age was analysed both as a continuous and categorical variable; in the latter case was recoded into 4 groups: under 30, >30-≤45, >45-≤60 and >60-≤75 years old. Donation is allowed after one month after the end of COVID-19 symptoms in our country.

Kolmogorov-Smirnov test was performed on each continuous variable to contrast normality. None of them followed normal distributions, therefore a non-parametric Mann-Whitney U test was performed. To contrast the independence of categorical variables, Pearson’s Chi-square and Fisher’s exact test were carried out. All tests were calculated with a confidence level of 0.05.

The Biobank is included in the National Registry of Biobanks (RD17 / 16/2011) with the number B.0000264. The institution holds an ISO 9001: 2015 certification endorsing our granting of safety and traceability of any human biological sample we distribute, always following Spanish and European rules on human samples and data protection management.

This study was conducted according to national regulations, institutional policies and the tenets of the Helsinki Declaration. It was approved by the Valladolid Health Area Drug Research Ethics Committee, on June 11th, 2020 with the reference number BIO-2020-93. Included donors gave written consent to participate in Biobank research activities. Privacy rights were always observed.

## Results

A total of 2421 randomized donations (either whole blood, plasmapheresis or platelet apheresis) from 1,107 repeat donors aged 46+/-12 were tested for total anti-SARS-CoV2 antibodies recognizing N protein (Table 1). 673 (15.4%) donations were positive. 71% of donors were convalescent plasma donors.

**Table 1:** Baseline characteristics of repeat donors.

		n	%
Blood group	A	512	46.3%
	B	72	6.5%
	O	478	43.2%
	AB	45	4.1%
Gender	Male	772	69.7%
	Female	335	30.3%
Age group	under 30	131	11.8%
	30–45	337	30.4%
	45–60	533	48.1%
	60–75	105	9.5%
	older than 75	1	0.1%

**Fig 1:**
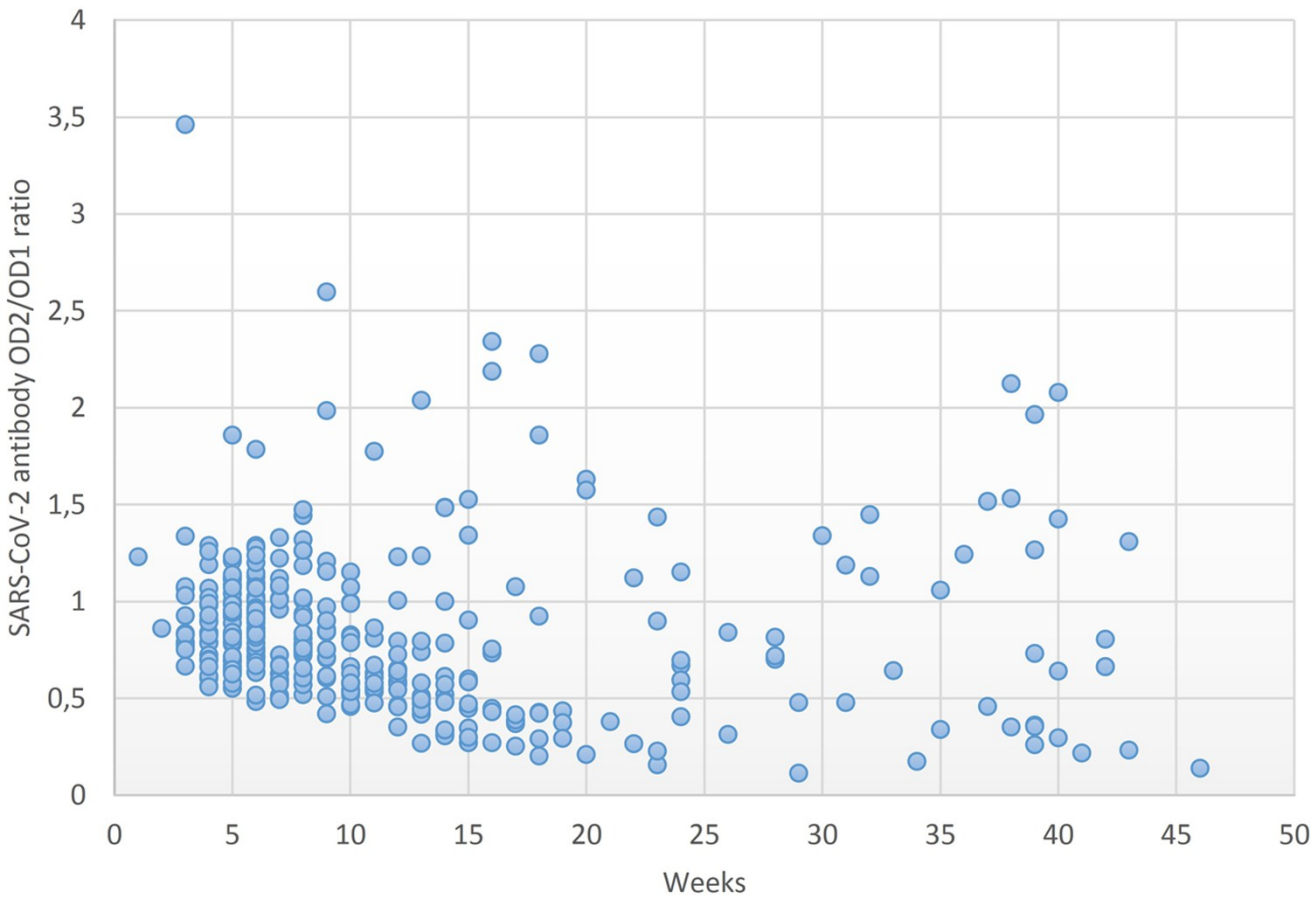
OD ratios along time. OD ratios from total immunoglobulins recognizing SARS-CoV-2 N protein of second over first donation are represented vs time lapse between them (weeks). OD: Optical density.

250 donors were positive at least once and included in the study provided a second donation after the positive one was tested. Their 588 donations underwent further analysis. 62 donors had more than 2 positive donations. The minimum time from symptoms’ relapse to blood donation is one month (a bit longer than 4 weeks). The average time gap between samples was 12.4 weeks (1–46, SD = 9.65). The longest period lapse between the first and last donation was 46 weeks. Ten donors could be followed up for longer than 40 weeks (that is 44 weeks after infection resolution), and all of them remained positive after this time (Fig 1).

**Fig 2:**
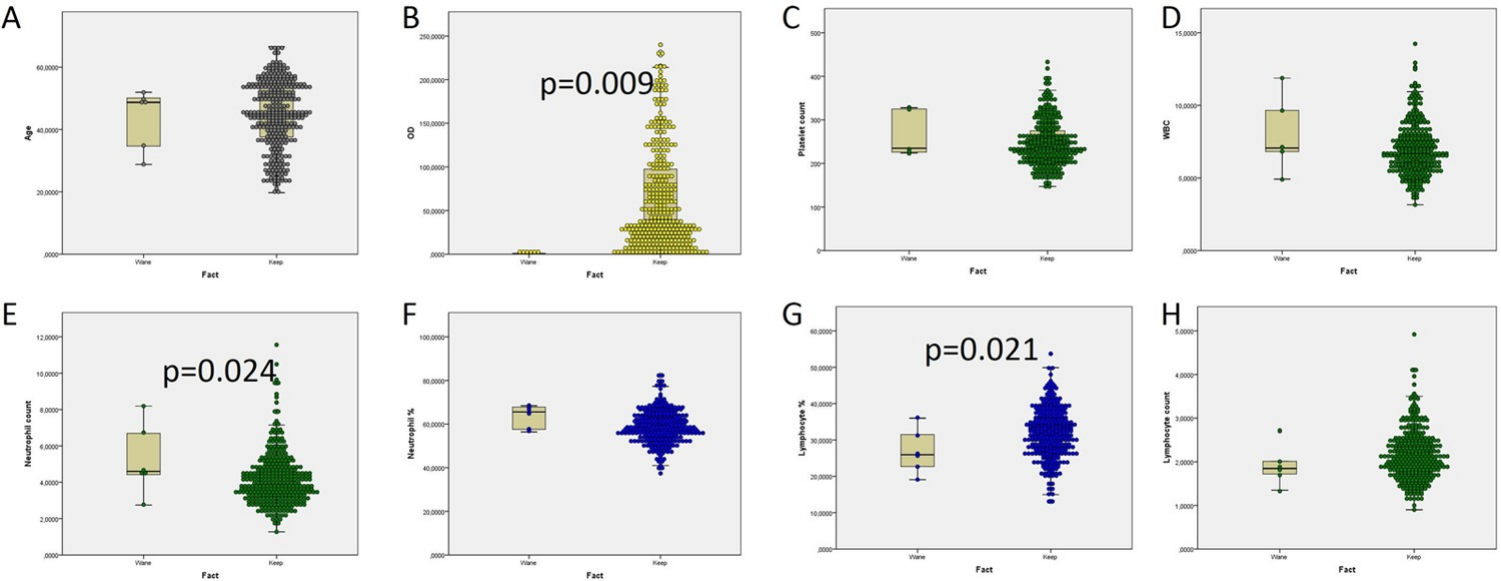
Analysis of age and haemogram. Comparison of age, OD from total immunoglobulins recognizing anti SARS-CoV-2 N protein, and haemogram in donations from antibody-keeping and antibody-waning donors. Wane: A positive donor becomes negative. OD: Optical density.

**Fig 3:**
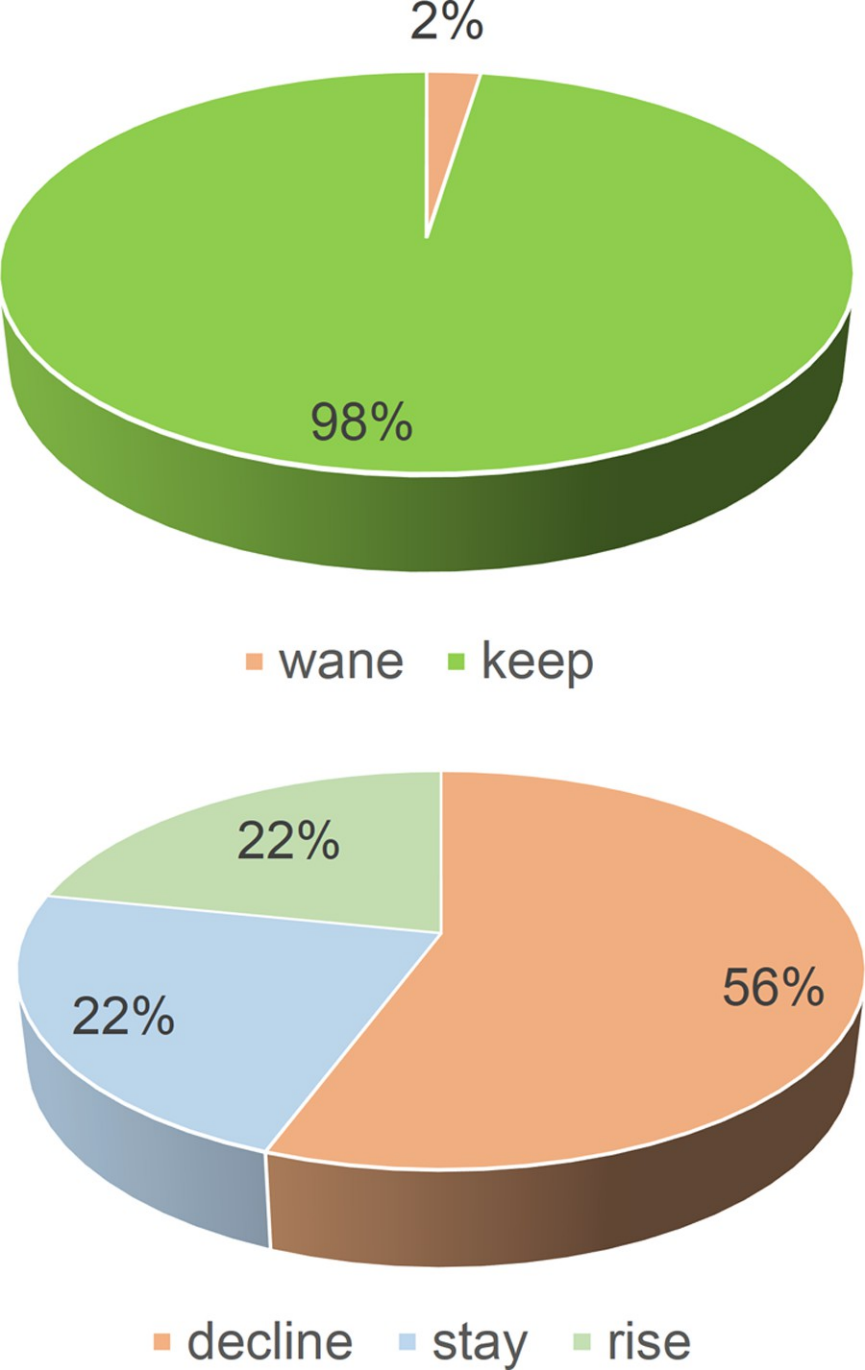
Outcomes of anti SARS-CoV-2 total antibodies. Proportion of donors keeping or waning total immunoglobulins recognizing anti SARS-CoV-2 N protein and donors whose total immunoglobulins recognizing anti SARS-CoV-2 N protein will decline, remain the same or rise. Wane: Positive donor becomes negative; decline: OD under 90% of the previous one; stay: OD +/-10% of the previous one; rise: OD over 110% of the previous one.

Those donors who would wane exhibited significantly lower initial ODs as analysing total antibodies of their first donation (1.5 vs. 74.4; p = 0.009, Fig 2B). Most (97.6%) donors kept positive their total antibodies against SARS-CoV-2. Out of the 250 initially positive donors, only 6 (2.4%) became negative at any moment of their follow-up (Fig 3). They were two males in their fifties and four women, two in their fifties again and two younger ones (Table 2). No other wane events were found.

**Table 2:** Characteristics of donors loosing anti SARS-CoV-2 antibodies.

Sex	n	Age group	Blood group	OD 1 (mean(SD))	OD 2 (mean(SD))	Weeks (mean(SD))
Male	2	≥45 and <60	A, O	1.51 (0.59)	0.55 (0.05)	12 (1.41)
Female	1	under 30	A	1.05 (-)	0.88 (-)	26 (-)
	1	≥30 and <45	A	1.08 (-)	0.56 (-)	14 (-)
	2	≥45 and <60	O	1.06 (0.02)	0.72 (0.23)	20 (8.49)

Weeks: Time gap between positive and negative donations (weeks); OD1: Optical density of first positive donation, OD2: Optical density of consecutive donation to the first positive one.

No relationship with age or blood group was found.

**Fig 4:**
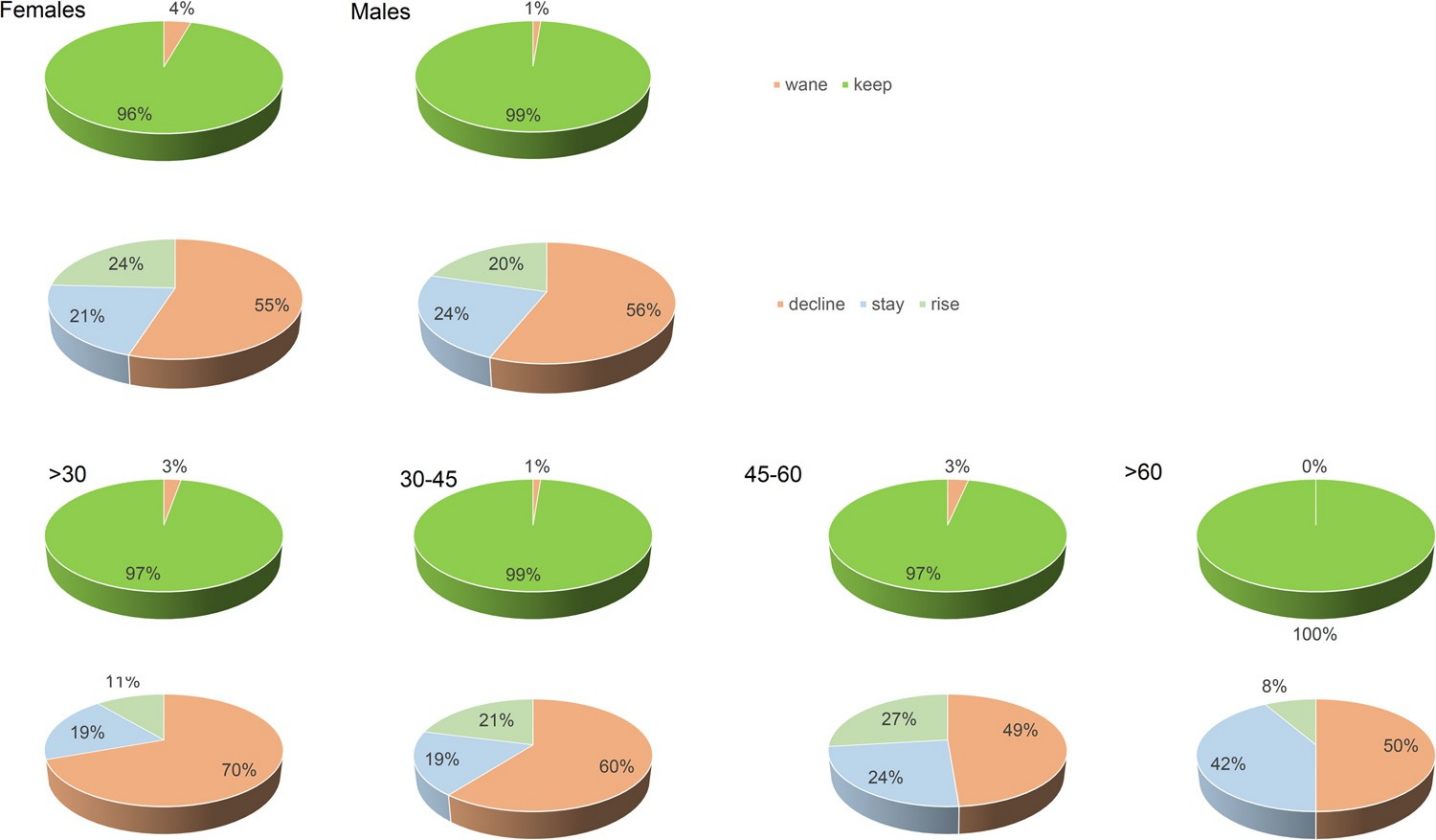
Sex and age relationship to outcomes of anti SARS-CoV-2 total antibodies. Proportion of donors keeping or waning total immunoglobulins recognizing anti SARS-CoV-2 N protein and donors whose total immunoglobulins recognizing anti SARS-CoV-2 N protein will decline, remain the same or rise, layered by sex and by age group. Wane: Positive donor becomes negative; decline: OD under 90% of the previous one; stay: OD +/-10% of the previous one; rise: OD over 110% of the previous one.

Waning was more frequent in women, not significantly, but as a trend (OR = 3.56; CI (0.64–19.8);p = 0.118). Rising OD was less frequent within the under 30 group, not significantly, but as a trend (OR = 0.4; CI (0.13–1.21); p = 0.153) (Fig 4, S2 Table in S1 File).

Haemogram parameters (leukocytes, neutrophils, lymphocytes, monocytes, eosinophils, basophils, haematocrit and haemoglobin) from each donor were tested for their relationship with antibody waning (S3 Table in S1 File, Fig 2). In those donors whose antibodies would wane, lymphocyte percent was significantly lower (24.1% vs. 31.8%; p = 0.021) conversely to neutrophil percent (65.9 vs 58.8; p = 0.024).

**Fig 5:**
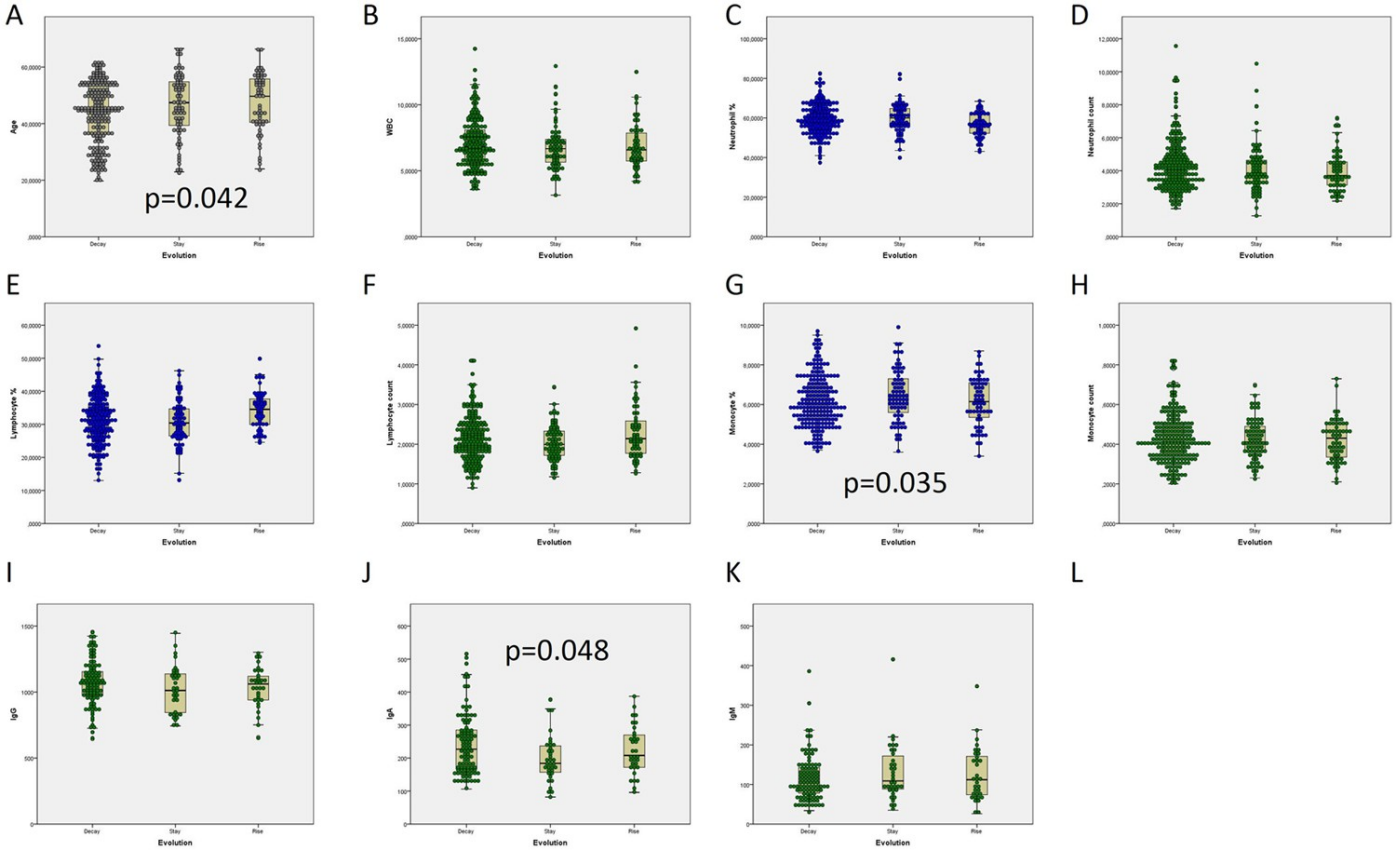
Analysis of age, haemogram and immunoglobulins in donations. Comparison of age, haemogram, and total, non-specific immunoglobulins in donations from donors whose total immunoglobulins recognizing anti SARS-CoV-2 N protein will decline, remain the same or rise, as regarding their second donation. Decay: OD under 90% of the previous one; stay: OD +/-10% of the previous one; rise: OD over 110% of the previous one.

ODs decayed as considering consecutive pairs of donations in 204 ones from 140 donors (56.0%, mean lapse between analysis, 9.5 weeks), rose in 61 pairs of donations from 54 donors (21.6%, mean lapse 11 weeks) and stayed +/-10% in 75 pairs of donations from 56 donors (22.4%, mean lapse 5.43 weeks) donors (Fig 3). Donors whose ODs decayed were significantly younger (45.0 vs 47.4 or 49.7 years old; p = 0.042, Fig 5A) and had fewer monocytes (6.0 vs 6.35 or 6.30 cells*103/μL; p = 0.035, Fig 5G). The ones whose OD due to anti SARS-CoV-2 total antibodies stayed had lower IgA (184.0 vs 208.0 or 227.0 mg/dL; p = 0.048, Fig 5J).

**Fig 6:**
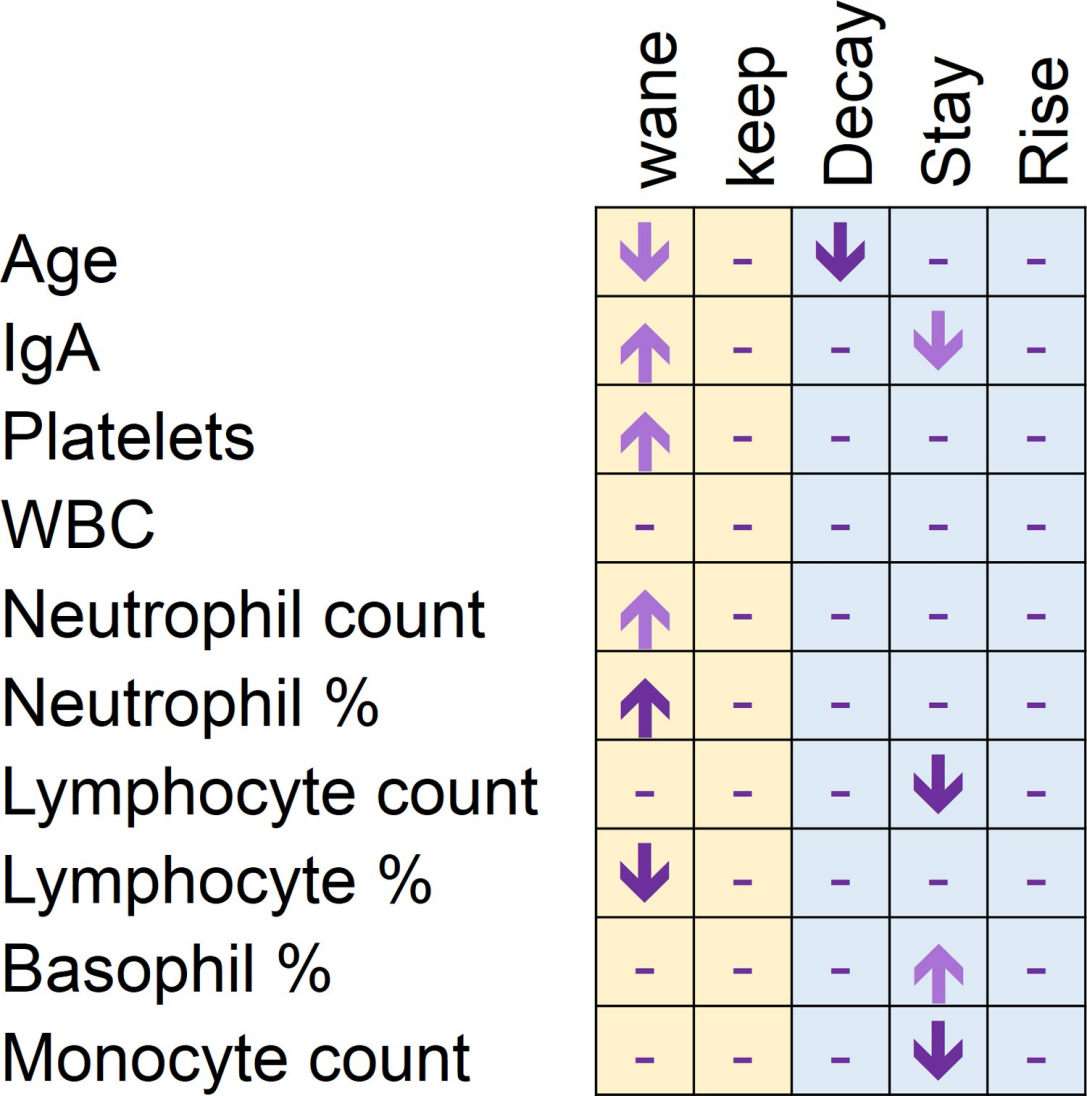
Summary of changes in in anti SARS-CoV-2 total antibodies related to age and hematological parameters. Downshift arrows indicate that low values of the parameter in rows are related to the fact in columns. Wane: Positive donor becomes negative; decay: OD under 90% of the previous one; stay: OD +/-10% of the previous one; rise: OD over 110% of the previous one. Light-coloured arrows represent statistic trends (0.05 < p <0.2).

A graphical summary of findings is represented in Fig 6.

## Discussion

Some reports at the beginning of pandemics pointed out that up to 40% of asymptomatic cases could lose their antibodies after 6 weeks from acute infection [16], but our data don’t support that statement. More recent studies [3,7] demonstrate that durable serum antibodies would be granted by long-lived plasma cells. Our study confirms that most individuals would keep their antibodies for at least 16 weeks. The 10 individuals that could be followed up 40 weeks (44 weeks after symptom relapse) were all still positive. Our results agree as well with others reporting a high percentage of individuals (above 75% or up to 90%) keeping antibodies positive for at least 9 months or a year even when a titer decline is noticeable and regardless of disease severity [7,15]. Donation is not allowed for asymptomatic positives until one month after negative testing nor for COVID-19 symptomatic cases, then 4 weeks should be added to follow-up time, which would mean at least 50 weeks after contagion, regarding our longest one. There is little literature on SARS-CoV-2 antibody waning. Antibodies recognizing N protein of SARS-CoV-2 have been elsewhere reported to wane in the post-infection phase, while those against the S protein would persist over time [16]. Additionally, the antibody response would be robust in patients with severe infection and weaker in those with mild infection or asymptomatic individuals. One of our limitations is that we cannot recall how many of our donors developed severe symptoms. Another one would be, that active blood donors are presumably in good health condition, and conclusions might not be extrapolated to people with other health conditions.

A little more than one half (56.0%) of our donors exhibit a decay in their OD from total immunoglobulins anti-SARS-COV-2 assay, although most of them remain positive. Specific immunoglobulins would become detectable within 5–7 days post-infection, and seroconversion would happen after 10–14 days. Detectable levels of neutralizing antibodies against SARS-CoV-2 have been shown to start declining within three months of infection, especially among mild and asymptomatic cases [17]. Previous studies reported that it is possible that the low titers found in some individuals still provide them protection against SARS-CoV-2. This decay can be part of a typical response following an acute viral infection, which usually peaks at 21–28 days and then begins waning. Logically, highest titers remain longer detectable, and those who first wane are the ones with lower ODs [18], accordingly to our data as well. The initial degree of antibody response would therefore be predictive of subsequently waning/decline. Either a decrease or loss of antibodies to SARS-CoV-2 would mean a weaker or slower immune response to infection, but there is no evidence of the amount of circulating antibodies that are necessary to provide protection against SARS-CoV-2 infection, disease, hospitalization, ICU admission, or death.

Antibody waning would be independent of the blood group in our cohort. Specific anti-SARS-CoV-2 immunoglobulin loss would be a rare event slightly more frequent in women, and decay would be more frequent in younger donors. Mature age would determine a longer antibody half-life [19]. Ageing is one of the most important determinants in COVID-19 severity [20,21], and it would have a role as well in humoral response keeping. The oldest patients are known to show both more severe forms of the disease and higher neutralizing activity indeed moderate correlations between age and neutralization have been reported, together with cytokine correlations [6]. Larger cohorts and replication analyses should be performed to determine whether an age-titer correlation exists because of the low rate of waning cases. An ageing effect cannot be discarded, but the low number of waning cases and the fact that none of them was older than 51 keeps us from further ascertainments, even when it points out a very interesting topic for future analyses.

Lower lymphocyte and higher neutrophil percentage might indicate future antibody waning. High neutrophil/lymphocyte ratios are somehow related to impaired innate and adaptive immune responses with a high inflammatory component and cytokine storm [22]. Our findings would reinforce the hypothesis of a not so robust response that would weaken in the medium term.

Some literature describes the correlation of neutralizing antibodies with specific IgG and total IgG within a series of cases that required hospitalization. We did not find any correlation of total antibodies OD with IgG, IgA or IgM, but IgA was lower in those donors of our series that would wane [23]. Total, non-specific, high IgA together with higher neutrophil and platelet count and lower neutrophil count might precede antibody waning as well. It is well known that several infectious agents such as malaria have prothrombotic effects, which happen as well in bacterial sepsis [24]. It has been elsewhere reported an elevation of platelets and their activation, and a lower number and activity of neutrophils in COVID-19 cases irrespective of their severity [25] accordingly to our data.

Secreted IgA is known to play a key role in SARS-CoV-2 immunity, but very little is known on circulating IgA [26]. IgG in combination with IgM and IgA would have greater neutralization capacity than IgG alone. A broader repertoire of antibodies correlates with a stronger SARS-CoV-2 neutralization [27]. Initial IgA production by plasmablasts declines quickly, whereas IgA-producing plasma cells can persist for years in mucosae [28]. It might explain that individuals mounting a strong IgA response would seem to lose antibodies, but it will be interesting to check out whether salivary IgA remains positive or not, so far IgA antibodies are the major component of the neutralizing antibodies developed in response to SARS-CoV-2 infection [29]

In conclusion, we confirmed our main hypothesis: Most donors keep their total immunoglobulins anti-SARS-CoV-2 positive for at least 16 weeks after symptoms relapse. We found as well that lower lymphocyte counts and higher neutrophils would help predict future negativization of antibodies. Last but not least, ageing might have a protective effect against antibody waning but, given the small number of cases that become negative, more studies, or larger series would be needed to confirm these relationships.

## Supporting information

S1 File (XLSX)
